# Differences in health care seeking behaviour between rural and urban communities in South Africa

**DOI:** 10.1186/1475-9276-11-31

**Published:** 2012-06-12

**Authors:** Marinka van der Hoeven, Annamarie Kruger, Minrie Greeff

**Affiliations:** 1 Africa Unit for Transdisciplinary Health Research (AUTHeR) and Centre of Excellence for Nutrition (CEN), Faculty of Health Sciences, North-West University, Potchefstroom Campus, Private Bag x6001, Potchefstroom, 2520, South Africa; 2 Africa Unit for Transdisciplinary Health Research (AUTHeR), Faculty of Health Sciences, North-West University, Potchefstroom Campus, Private Bag x6001, Potchefstroom, 2520, South Africa

**Keywords:** Accessibility, Health care seeking behaviour, Quality, Rural community, Urban community

## Abstract

**Objective:**

The aim of this study was to explore possible differences in health care seeking behaviour among a rural and urban African population.

**Design:**

A cross sectional design was followed using the infrastructure of the PURE-SA study. Four rural and urban Setswana communities which represented different strata of urbanisation in the North West Province, South Africa, were selected. Structured interviews were held with 206 participants. Data on general demographic and socio-economic characteristics, health status, beliefs about health and (access to) health care was collected.

**Results:**

The results clearly illustrated differences in socio-economic characteristics, health status, beliefs about health, and health care utilisation. In general, inhabitants of urban communities rated their health significantly better than rural participants. Although most urban and rural participants consider their access to health care as sufficient, they still experienced difficulties in receiving the requested care. The difference in employment rate between urban and rural communities in this study indicated that participants of urban communities were more likely to be employed. Consequently, participants from rural communities had a significantly lower available weekly budget, not only for health care itself, but also for transport to the health care facility. Urban participants were more than 5 times more likely to prefer a medical doctor in private practice (OR:5.29, 95% CI 2.83-988).

**Conclusion:**

Recommendations are formulated for infrastructure investments in rural communities, quality of health care and its perception, improvement of household socio-economical status and further research on the consequences of delay in health care seeking behaviour.

## Introduction

Designing health care policies and programmes requires knowledge about health care seeking behaviour, so that possible difficulties with early diagnosis and effective treatment can be identified and so that appropriate interventions can be implemented. Early recognition of symptoms, presentation to health care facilities and compliance with effective treatment can reduce morbidity and thereby mortality [1,2]. In addition, successful adherence to health care programmes is determined by the interactions of (ill) people with health care systems [3]. South Africa is currently experiencing an epidemiological transition in which it has to carry the so-called quadruple burden of disease, which presents a great challenge for designing health care policies and programmes. This quadruple burden refer to an increased burden of chronic diseases, maintenance of poverty-related diseases, injuries and to a rise in infectious diseases associated with HIV and Acquired Immunodeficiency Syndrome (AIDS) at the same time [4]. Together with this epidemiological transition, South Africa is also experiencing a nutrition transition and a demographic transition. The nutrition transition encompasses a shift from a high prevalence of under-nutrition to diet-related non-communicable diseases. The shift from a pattern of high fertility and high mortality to one of low fertility and low mortality is referred to as the demographic transition [5]. The process of shifting from a low-income country towards a middle-income country is associated with both negative and beneficial effects. Hence, the association between socio-economic status and health status is becoming more complex as a result of the slow growth of the economy and the ongoing urbanisation in South Africa. Chronic diseases are not only associated with persons of high socio-economic status; nor are infectious diseases only prevalent among persons of low socio-economic status. Nevertheless, infectious diseases are primarily prevalent among the poor (black) community. This relation is, however, not inevitably present within a racial group. In addition, the differences between urban and non-urban residents of the African majority in terms of their health status can be partly explained by the distinct effects of urbanisation on health [6]. Franzini et al. [7] also indicated that individual self-rated health is affected by a complex interplay of neighbourhood characteristics and that neighbourhood poverty is an important contributor to health status. This poverty intensifies negative social processes at the neighbourhood level, such as disorder and racism, which in turn leads to poorer self-rated health [7].

The different characteristics of urban and rural areas in South Africa contribute to a difference in terms of self-rated health [8] and possibly also in health care seeking behaviour. Health care seeking behaviour will be influenced by the individual self, diseases, and the availability and accessibility of health services. Dependent on these determinants and their interactions [9,10], health care seeking behaviour is a complex outcome of many factors operating at individual, family and community level. Therefore, health care seeking behaviour in an urban and rural African population was compared in order to formulate recommendations which will assist with the design of health care policies and programmes. A cross sectional design was followed on the baseline data of the PURE-SA-NWP study.

## Methods

This study on health care seeking behaviour was part of the South African division of the Prospective Urban and Rural Epidemiological (PURE-SA) study focusing done in the North West Province (NWP) of South Africa on (changes in) lifestyle, risk factors and non-communicable diseases over a period of twelve years (2005–2017). This study on health care seeking behaviour was designed as a case study on a representing sample of the 1999 participants in the PURE-SA-NWP study.

### Research population

Rural and urban communities which represent four different strata of urbanisation in the North West Province, South Africa and where history predicted relative stability regarding migration, were identified for the longitudinal PURE-SA study. The four communities were all part of the Setswana culture in the North West Province and were selected to ensure that the focus would be on the possible differences between urban and rural communities and not between cultures. Two urban communities in and around Potchefstroom were chosen; the first was selected as an established urban community from the established part of the township adjacent to Potchefstroom and the second as an informal community from among the informal settlements that surround the established community. Two rural communities were chosen: the first is situated 450 km west of Potchefstroom on the highway to Botswana and the second is a deep rural community situated 35 km northeast from the first rural community. The second rural community is only accessible by gravel road. Both rural communities are still under tribal law and have the same chief. All the participants of the PURE-SA study (N = 1999) formed the population for this study. The inclusion criteria for participating in the PURE-SA study were: [1] living in one of the above-mentioned communities; [2] regarding oneself as being healthy (not being aware of any disease); [3] not taking medication for a chronic disease; [4] being older than 35 years; [5] not being pregnant; and [6] not being inebriated (while measurements were taken).

### Sampling

Sampling of subjects in this health care seeking behaviour study followed a stratified random selection from the 1999 participants of the PURE-SA study. Structured interviews collecting quantitative as well as qualitative data, based on a data saturation approach were used. A total of 224 participants were interviewed of which 18 persons were excluded in the data analyses because of incomplete data. Of the 206 participants, 125 were urban inhabitants and 81 were rural inhabitants.

### Research measuring instrument

The questionnaire used in this study consisted of 53 multiple-choice and open-ended questions. There were 11 qualitative questions on general demographic characteristics, occupation and income, housing, impact of several diseases and (access to) health care. The questions on health status (n = 2) were obtained from the Medical-Outcomes-Study (MOS) 36-item short form survey instrument. The score of general health was based on five items, namely ‘In general would you say your health is…´, ‘I seem to get sick a little easier than other people’, ‘I am as healthy as anybody I know’, ‘I expect my health to get worse’ and ‘My health is excellent’ [11,12]. The reliability of these five items together was considered acceptable, because the Cronbach’s alpha was 0.78 [13]. Two rounds of face and content validity testing of the total questionnaire and to acquainting for cultural sensitivity were conducted on staff members of Setswana origin. Furthermore, it was pre-tested on members in the community who served as field workers to the PURE-SA study, and on ten participants in the PURE-SA study who were not included in this study.

### Data collection

Data was collected by means of structured face-to-face interviews conducted by 14 fieldworkers (seven in the urban communities, seven in the rural communities) during a two-month period. The fieldworkers received training prior to conducting the interviews. The researchers initially accompanied each fieldworker for up to three interviews, to ensure the correct procedure. The interviews were conducted in the mother tongue of the participant. The participants were interviewed without disturbance in the privacy of their own homes.

### Statistical analysis

The data was entered and analysed by means of Statistical Package for Social Science 15.0 for Windows software. After data cleaning, the dataset was tested for normality with the Shapiro-Wilk test and was found to violate the assumption of normality for several variables, which could not be improved by transformation. Therefore, several non- parametric methods (Mann–Whitney U test, Kruskal-Wallis test and Chi-square test) were used to analyse the data. Correlation was expressed as the non-parametric Spearman’s Rank Order Correlation (rho). A p value of <0.05 was considered significant (confidence interval (CI): 95%).

### Ethical considerations

Ethical consent for this study was granted as an addendum to the PURE-SA study approved by the ethical committee of the North-West University (Potchefstroom Campus), South Africa (number: 04 M10). Permission for the study to be conducted was also obtained from the Provincial Department of Health of the North West Province, the local government authorities of each town as well as the tribal chiefs in the rural communities. Participants received a written and an oral explanation of the study and of the purpose of the study in their mother tongue. Before participation in the study or in any follow-up action, all participants gave informed and written consent. Participation was voluntary and participants could withdraw at any time without consequences. Personal details and the collected data were stored completely anonymous.

## Results

### Socio-demographic characteristics

Table 1 summarises the socio-demographic characteristics of the participants of this study, separately for inhabitants of urban and rural communities. This data made it clear that significantly more females than males participated in both urban and rural communities (both p < 0.0005 [not shown in the table]). There was no difference between the urban and rural communities in this respect. The urban participants in this study were older and more of them were employed than participants of in the rural communities (respectively p = 0.010 and p = 0.002). Furthermore, the urban participants’ available weekly budget was in general bigger (p = 0.029), particularly in the case of employed participants (p < 0.0005). There was also a significant difference between the sources of this income for the two groups (p < 0.0005). The major source of the available weekly budget for urban participants was labour, whereas diverse grants were the main source of income for rural participants. In the rural communities, the mean income from labour was R 46 per week and the mean income from diverse grants was R 125 per week.

**Table 1:** Socio-demographic characteristics of participants

	**Urban (N = 125)**		**Rural (N = 81)**		***χ***^***2***^	***p***	
	***N***	***(%)***	***N***	***(%)***			
Gender					3.462	0.063	
· Male	58	(46.4)	27	(33.3)			
· Female	67	(53.6)	54	(66.7)			
Occupation at this moment (N = 204)					9.932	**0.007**	
· Employed	30	(24.0)	8	(9.9)			
· Unemployed	59	(47.2)	55	(67.9)			
· Pensioner	36	(28.8)	18	(22.2)			
Source of budget (N = 180)					45.795	**0.000**	
· Labour	45	(41.3)	7	(9.9)			
· Pension	35	(32.1)	18	(25.4)			
· Partner and/or children	19	(17.4)	11	(15.5)			
· Family and/or friends	5	(4.6)	7	(9.0)			
· Grants^†^	5	(4.6)	28	(39.4)			
	*Mean±SD (range)*		*Mean±SD (range)*		*U*	*z*	*p*
Age^‡^	53.5±11.0 (37–80)		49.5±9.3 (37–76)		3989.0	−2.57	**0.010**
Weekly available budget^§^ (N = 174)	185±122.3 (0–800)		128±75.0 (0–235)		2779.5	−2.18	**0.029**
· Employed (N = 36)	271±132 (50–800)		62±33 (30–100)				**0.000**
· Unemployed (N = 88)	124±132 (0–500)		112±31 (0–235)				0.370
· Pensioner (N = 50)	192±50 (50–300)		202±31 (90–200)				0.627

^†^ Grants included grants for children and disability grants.

^‡^ Age in years; ^§^Weekly available budget in South African Rand.

### Health care seeking behaviour

#### Health beliefs

Participants were asked to describe the impact of six diseases (cancer, diabetes, heart problems, HIV or AIDS, hypertension and tuberculosis) on a person’s daily life. HIV or AIDS was identified as the disease with the biggest impact according to both urban and rural participants. A significant difference was found between urban and rural communities in terms of their rating of how diabetes, tuberculosis, cancer and heart problems impact on daily life. Participants in urban communities were more likely to rate the impact of diabetes (p = 0.021), cancer (p < 0.0005) and heart problems (p = 0.004) as large, whereas participants in rural communities were more likely to rate the impact of tuberculosis as large (p = 0.031). 76.5% of the participants (N = 204) agreed that people need medical help when they are ill or do not feel well. Of these participants, 23.1% added that the person seeking medical help must be very sick/ill. There was a significant difference between urban and rural participants’ responses to these statements. Participants in rural communities were more likely to agree with the first statement (p = 0.027), whereas urban participants more often added that someone who seeks medical help must be very sick/ill (p = 0.053). Furthermore, significantly more rural participants than urban participants expressed the opinion that people need medical help when they experience pain (p = 0.002).

### Health status

The urban participants rated their health in general (on a scale of excellent [1] to poor [5]) better than the rural participants (χ2 = 6.559; p = 0.015). There was also a statistically significant difference (p = 0.045) in the rating of health across different age groups in urban communities. This was reflected in rural communities (p = 0.053) Table 2.

**Table 2:** Health status

	***Urban (N = 125)***		***Rural (N = 81)***		***χ***^***2***^	***p-value***
	***Mean±SD (range) or N (%)***					
Rated health in general			2.8±1.51 (1-5)		6.052	**0.049**
	3.3±1.39 (1-5)					
Ill during one or more days in the last thirty days					2.572	0.276
· Agree	59	(47.2)	32	(39.5)		
· Disagree	66	(52.8)	49	(60.5)		
Severity of illness	6.7±2.93 (1-10)		5.1±2.21 (1–10)		22.711	**0.007**
Helpfulness action to feel better					9.435	**0.024**
· Disagree	2	(3.9)	7	(25.0)		
· Agree	47	(92.2)	21	(75.0)		
· A little	2	(3.9)	0	(0.0)		

Urban and rural participants in the age group 55 to 64 years old rated their health the best. Within the urban community, the age group of 65 years and older older rated their own health the poorest, but among rural participants it was the age group of 35 to 44 years who rated their own health the poorest. Urban and rural inhabitants did not differ significantly in terms of their current rating of their health, compared to how they rated their health one year earlier: 28.5% of the rural participants and 34.6% of the urban participants rated their current health about the same as one year before, whereas 38.4% and 25.9% respectively rated their current health somewhat/much better than a year before, and 27.2% and 39.5% respectively rated it somewhat/much worse. This variable had a strong positive association with health ratings for both urban and rural communities (respectively r = 0.418, p < 0.0005 and r = 0.419, p < 0.0005). Participants, who rated their health as excellent, were more likely to report that their health had improved compared to one year ago. There was no significant difference between the urban and rural communities in terms of how participants rated their health now, compared to one year earlier; and the mean difference was respectively 0.66 (SD 1.2) and 0.79 (SD 1.1) on a scale of excellent [1] to poor [5].

Almost half the urban (47.2%) and rural (39.5%) participants felt ill for one or more days in the preceding 30 days. Urban and rural participants did not differ significantly with regard to feeling sick on one or more days in the preceding 30 days and a medium negative correlation with rated health was found for both urban (r = −0.271, p = 0.002) and rural (r = −0.246, p = 0.028) communities. Both urban and rural participants who felt sick during one or more days in the preceding 30 days rated their health worse than the other participants. Furthermore, urban and rural participants rated the severity of their illness significantly different (χ2 = 22.711; p = 0.007); respectively with a mean of 6.7 (SD 2.93) and 5.1 (SD 2.21) on a scale of 0 (not severe) to 10 (severe). Of the participants who felt sick, 86.4% of the urban and 87.5% of the rural inhabitants did something which could help them to feel better. Most urban respondents were able to treat themselves successfully (92.2%), whereas rural inhabitants were less likely to do so (75.0%) (p = 0.015).

### Health care utilisation

74.8% of urban and 75.6% of rural participants were of the opinion that, they had sufficient access to health care. There was no significant difference in this regard between participants living in rural communities and those living in urban communities. Reasons for not having sufficient access to health care included transport/distance to health care facilities, financial constraints, and problems with the service. Problems with the service included provision and availability of medication, number and quality of the staff, facilities (including equipment), service hours and capacity (ability to accomodate all the patients within a reasonable time). There were no significant differences in this regard between rural and urban communities.

Table 3 shows which health care provider was preferred by participants. Urban and rural participants differed significantly with regard to their preference for a health care provider (p = 0.001) (Table 3). Inhabitants of urban communities preferred to visit a private medical doctor if there were no restrictions such as lack of money or transport, whereas rural participants preferred to visit a health clinic. Participants gave different explanations for their preference (Nurban = 99; Nrural = 65). Most urban participants referred to financial constraints (44.4%) and to the expertise of the health care provider (42.4%), whereas most rural respondents referred to treatment (availability and quality) (80.0%) and financial constraints (58.5%). Participants could provide more than one reason for their responses.

**Table 3:** Preferred health care provider and reasons for visit ( in per cent)

	**Urban**	**Rural**
Preferred health care provider	N = 125	N = 81
· Private medical doctor	50.4	14.8
· Health clinic	31.2	71.8
· Traditional healer	5.6	9.9
· No (clear) preference	12.8	3.5
Reasons to visit a private medical doctor	N = 120	N = 52
· When a person is sick/ill or not feeling well^1^	48.8	71.5
· When a person is very sick/ill or not feeling well at all	21.5	7.6
· When a person has pain	7.0	1.2
· When health clinic or self-medication did not improve the condition	9.3	5.8
· When I have enough money	1.7	0.6
Reasons to visit a health clinic	N = 122	N = 77
· When a person is sick/ill or not feeling well^*^	48.4	68.8
· When a person is very sick/ill or not feeling well at all	7.4	7.8
· When a person has pain	4.9	16.9
· To fetch monthly treatment	32.8	10.4
· No money (e.g. for a private doctor)	10.7	7.8
Reasons to visit a traditional healer	N = 40	N = 25
· When Western science does not improve the condition^†^	32.5	16.0
· When I have problems/issues in the social part of my life	37.5	32.0
· When I have physical problems	22.5	28.0
· When I suspect witchcraft	2.5	16.0

^*^ Included ‘When a person is very sick/ill or not feeling well at all.

^†^ Western science: medical doctor, health clinic and/or regular treatment.

The number of visits to a private medical doctor in the preceding four weeks was not significantly different for urban and rural participants. A small number of urban respondents (3.2%) indicated that they never visit a private medical doctor, as opposed to 35.8% of the rural respondents. Most urban participants (82.4%) had not visited a private doctor in the previous four weeks, 12.8% only once and 4.8% two or more times. This pattern was similar for rural inhabitants, where the figures were respectively 80.2%, 14.8% and 5.0%. Table 3 summarises participants’ motivation for visiting a private medical doctor. The motivation most frequently offered by both urban and rural participants is ‘when a person is sick/ill or not feeling well’, although urban inhabitants more often added that the person needs to be very sick/ill or not well at all. The health clinic was visited more often. The first and the second quartile were the same for urban and rural inhabitants, respectively 0 and 1 visit. The third quartile for the rural inhabitants was 2 visits and for urban inhabitants 1 visit indicating that Lliving in a rural community was associated with a higher number of visits to a health clinic (p = 0.002). A small number of both urban (2.4%) and rural (4.9%) participants never visited the health clinic. Almost half of the urban participants (46.0%) had not visited the clinic in the previous four weeks, 31.5% only once and 22.5% two or more times. Approximately a third of the rural inhabitants (32.1%) did not visit the clinic in this period, 19.8% only once, 27.2% twice and 20.9% three or more times. Table 3 summarises reasons for visiting a health clinic. Both urban and rural participants indicated that the main reason for visiting a health clinic was that a person was sick/ill or not feeling well. For urban participants the health clinic is also an important site for obtaining monthly treatment. A large number of participants, both urban and rural, reported that they never visit a traditional healer (respectively 67.2% and 69.1%), with no significant difference between urban and rural communities. Of the urban participants who did visit a traditional healer, 7.2% responded that they visited a traditional healer once or more (up to four visits) in the previous four weeks, compared to 12.3% of the rural participants (up to a maximum of three visits). Again, there was no statistical difference between urban and rural communities. Both urban and rural participants visited a traditional healer primarily to obtain help with social problems. Other reasons are summarised in Table 3.

Tables 4, 5 and 6 present the adjusted odds ratios for the preference of health care provider. The crude odds ratios have been adjusted for gender and age. Urban participants were slightly more likely to prefer a private medical doctor over a health clinic or traditional healer as preferred choice of health care provider than rural participants (OR = 0.17, p < 0.005) (Table 4). This was confirmed by the second model, indicating that urban participants were more than 5 times more likely to prefer a private medical doctor or traditional healer over a health clinic as preferred choice of health care provider than rural participants (OR = 5.29, p < 0.005) (Table 5).

**Table 4:** Logistic regression predicting likelihood of preference private medical doctor

	**B**	**S.E.**	**p**	**OR**	**95% C.I. for OR**	
					**Lower**	**Upper**
Rural–urban	−1.78	0.37	<0.005	0.17	0.08	0.35
Gender	0.23	0.32	0.48	1.25	0.67	2.34
Age	−0.01	0.02	0.71	0.99	0.97	1.02
Constant	1.95	0.80	0.02	7.05		

R^2^ = 0.51 (Hosmer & Lemeshow), 0.13 (Cox & Snell), 0.18 (Nagelkerke). Model χ^2^(3) = 29.57, p < 0.005.

**Table 5:** Logistic regression predicting likelihood of preference health clinic

	**B**	**S.E.**	**p**	**OR**	**95% C.I. for OR**	
					**Lower**	**Upper**
Rural–urban	1.67	0.32	<0.005	5.29	2.834	9.876
Gender	0.02	0.31	0.94	1.02	0.555	1.885
Age	0.01	0.02	0.36	1.01	0.985	1.044
Constant	−1.61	0.78	0.04	0.20		

R^2^ = 0.37(Hosmer & Lemeshow), 0.15 (Cox & Snell), 0.20 (Nagelkerke). Model χ^2^(3) = 33.91, p < 0.005.

**Table 6:** Logistic regression predicting likelihood of preference traditional healer

	**B**	**S.E.**	**p**	**OR**	**95% C.I. for OR**	
					**Lower**	**Upper**
Rural–urban	0.64	0.55	0.25	1.90	0.64	5.62
Gender	−0.69	0.56	0.21	0.50	0.17	1.49
Age	0.03	0.03	0.37	1.03	0.97	1.09
Constant	1.21	1.43	0.40	3.36		

R^2^ = 0.74 (Hosmer & Lemeshow), 0.02 (Cox & Snell), 0.04 (Nagelkerke). Model χ^2^(3) = 3.45, p = 0.25.

Participants who reported that they visited a certain health care provider were asked to described, their expectations of that provider. Participants could report more than one expectation per provider. Urban participants’ main expectations of their private medical doctor was to receive (the right) treatment (72.1%), to be examined (35.1%) and to receive help or to be healed (23.4%). Rural participants who visited a private medical doctor expected (the right) treatment (80.0%), to receive help or to be healed (30.0%) and to be examined (12.5%). Participants had similar expectations of the health clinic. (The right) treatment was an expectation among 86.3% of the urban participants and among 61.9% of the rural participants. 20.0% of the urban participants and 36.5% of the rural participants expected to receive help or to be healed, while. 68.0% of the urban participants expected to get traditional treatment from the traditional healer (muti and/or herbs); compared to 78.6% of the rural participants. In addition, 24.0% of the urban inhabitants expected to be healed or to receive help from the traditional healer, compared to 28.6% of the rural inhabitants.

## Discussion

The fact that more women than men participated in both urban and rural communities might be. because women are more likely to seek and use health care, possess greater knowledge about health, are compliant with a therapeutic regimen and monitor the health of others as well as their own health, [14], although not explicitly explored in this study. Significantly fewer people in rural communities were interviewed as data saturation was quicker reached indicative of more homogeneousity. The difference between employment rate in urban communities and that in rural communities, as found in this study, indicated that members of urban communities were more likely to be employed. This is confirmed by Banerjee et al. [15], who pointed out that unemployment has risen in South Africa since the first democratic elections in 1994, among several other reasons because there is a mismatch between places where jobs were available in the formal sector are and places where (unemployed) people live. Consequently, the available weekly budget for participants from rural communities was significantly smaller. The source of this income obviously differed for employed and unemployed participants. During the interviews, the researchers in the field probed for income from day jobs, especially in interviews with participants who considered themselves as unemployed. This still did not explain the unexpected difference between employed and unemployed rural participants with regard to their available weekly budget. The difference might be ascribed to factors such as that family members (e.g. adult children who are employed) regularly send money home to the unemployed family members. In rural communities, there were often more unemployed participants receiving grants than there were employed participants in paid work. In addition, the unemployed participants received more money in the form of grants than the employed earned through their labour. Most grants were Child Support Grants, which are provided by the government to ensure that caregivers of young children living in extreme poverty are able to access financial assistance in the form of a cash transfer to supplement, rather than replace, household income [16]. The review of the Child Support Grant in 2008 confirmed that among individuals who were eligible for the grant, caregivers in rural or informal urban areas were more likely to receive the grant than those in formal urban areas [16].

Participants living in urban communities rated their health significantly better than rural participants. Health status is linked to socio-economic status, and could therefore be related to the fact that unemployment is more prevalent among rural participants, who consequently have less money available to spend on good nutrition and health care. Although visits to governmental health clinics and (prescribed) medication at these facilities are free of charge in South Africa, transport to these services is not available for everyone, due to an absence of transport or a lack of money to pay for transport. This is especially the case in rural communities, where distances to health clinics can be relatively long [17]. Furthermore, there is a significant difference in terms of rated health across different age groups in urban and rural communities. Urban participants of 65 years and older and rural participants in the age group 35 to 44 years rated their health the poorest. The urban participants of 65 years and older possibly experience health problems associated with aging, whereas the health problems of the younger rural group could possibly be ascribed to the fact that the HIV prevalence among this age group is significantly higher than among the other age groups. The urban population is also older than the rural population, which could explain why the different age groups in the communities rated their own health differently. Almost half the urban and rural participants felt sick on one or more days in the preceding 30 days and both urban and rural participants who felt sick rated their health worse than other participants. Members of urban communities were likely to rate the severity of this illness higher, and most of them were able to treat themselves successfully, whereas rural participants were less likely to do so. Although medication is available for free at health clinics, there are often long queues, which makes going to the clinic a day trip. Urban communities have local pharmacies where medication can be purchased without a prescription, but rural areas lack these facilities.

Urban participants rated the impact of diabetes, cancer and heart problems on their daily lives larger than rural participants did. A possible reason why these diseases are reported more frequently by the older participants in urban communities is that these diseases better known among that age group. Effects of the epidemiological transition are also visible in these urban communities, and changes in lifestyle, including dietary and activity patterns, resulted in an increased incidence of non-communicable diseases. Increasing urbanisation also accounts for increasing levels of stress and a decline of the traditional social support systems [18]. Rural participants, in turn, rated the impact of tuberculosis on their daily lives larger. Tuberculosis had a higher prevalence in rural areas than in urban areas and therefore had a bigger impact on the daily lives of rural participants. Most participants in both urban and rural communities rated the impact of HIV or AIDS as large and participants in both communities indicated that its effect was worse than that of any other disease. The prevalence of HIV or AIDS was similar in urban and rural communities.

Urban and rural participants have similar expectations of the different health care providers: they expect (proper) treatment and want to be healed or to receive help. However, urban and rural participants have different patterns of utilisation of health care providers. Members of urban communities preferred to visit a private medical doctor, whereas rural participants preferred to visit a health clinic. Again, this was also related to the available weekly income, since a health clinic provides free care and a medical doctor in private practice does not. Urban participants considered the care provided by a private medical doctor, for which they must pay, as superior to that provided by a health clinic, mainly because the service was better. Rural participants reported that they had to be very ill before they would visit a private medical doctor. The main motivation for visiting a doctor in private practice or a health clinic was the same for most urban and rural participants, namely because they were sick/ill or not feeling well. Another important reason for urban participants to visit a health clinic is to obtain monthly treatment for non-communicable diseases, such as hypertension and diabetes.

A large number of respondents, both urban (67.2%) and rural (69.1%), indicated that they never visit a traditional healer. This is confirmed by Hirschowitz et al., who found that 33.3% of the African urban population and 30.7% of the African rural population of South Africa visit a traditional healer [19]. In contrast, Pinkoane et al. [20] found that an estimated 80% of the black population use traditional medicine. Approximately a third of this study population (urban and rural) visited a traditional healer primarily to address social problems. Urban participants in particular would visit a traditional healer when Western science did not improve their condition. Significantly, rural participants attached equal importance to social problems and to incidents of suspected witchcraft as reasons for visiting a traditional healer.

25.2% of urban participants and 24.4% of rural participants described access to health care as insufficient. Most urban and rural participants considered their access to health care sufficient, although 55.0% of the urban participants and 45.6% of the rural participants experienced difficulties with accessing health care. Both urban and rural participants experienced difficulties with regard to transport/distances to facilities, financial constraints, and/or the service provided by the health care facilities, not to the same extent. Dissatisfaction with the service included problems with the provision and availability of medication, the number and quality of the staff, facilities (including equipment), service hours and the capacity (ability to attend all the patients within a reasonable time). Small available budgets restrict the choice of health care providers, both in the case of urban and rural participants, and may also compel people to delay seeking health care [21]. Furthermore, because the public health service in South Africa is overburdened and under-staffed, waiting times are excessive and consultation times too short to be effective [19]. These findings confirm results of previous studies that financial considerations [22], perceived quality of a health care provider and the geographic location of the provider [23] are important criteria influencing an individual’s choice [24,25].

## Conclusions

The aim of this study was to explore whether there are differences between urban and rural communities in terms of health care seeking behaviour and what these possible differences encompass. This study identified several definite differences in health care seeking behaviour between members of urban and rural communities. The two groups differ in terms of their socio-economic characteristics, health status, health beliefs, prevalence of non-communicable and infectious diseases, and utilisation of health care. An important difference was the preference of health care provider; urban participants were more likely to prefer a medical doctor in private practice and rural participants were more likely to prefer a health clinic. Although most urban and rural participants consider their access to health care sufficient, they still experience difficulties with accessing the requested care. Based on the findings in this study, therefore, the following recommendations are made with regard to policy and practice:

(1) More infrastructure investments, including public transport, should be made to improve accessibility to health care, especially in rural areas.

(2) The quality of health care and the perception of this care should be improved. This includes the provision and availability of medication, the number and quality of the staff, facilities (including equipment), service hours and the capacity (ability to attend to all the patients within a reasonable time). This should be a priority, in rural areas in particular, where returning migrants with chronic diseases pose a significant challenge to the health systems [26].

(3) A transdisciplinary health team and multi-sectoral approach should be used to improve household socio-economical status, among others by addressing the structural problem of unemployment.

(4) Further research should be done on the consequences of delay in health care seeking behaviour, to decrease or possibly prevent the high costs of illness.

## Abbreviations

HIV, Human Immunodeficiency Virus; AIDS, Acquired Immunodeficiency Syndrome; PURE-SA, South African division of the Prospective Urban and Rural Epidemiology study.

## Competing interests

The authors declare that they have no competing interests. The authors declare that they have no financial or personal relationship(s) which may have inappropriately influenced them in writing this paper.

## Authors’ contributions

AK was the project leader, AK and MvdH were responsible for the project design. MvdH was responsible for the execution of the project and the statistical analyses. MvdH, AK and MG wrote the manuscript. All authors read and approved the final manuscript.

## References

[B1] Hausmann-Mueala S Muela Ribera J Nyamongo I Health-seeking behaviour and the health system response Disease Control Priorities Project (DCPP). Working Paper no.14; 2003 http://www.dcp2.org/file/29/wp14.pdf

[B2] World Health Organization Rapid assessment of health seeking behaviour in relation to sexual transmitted disease: draft protocol 1995 http://www.who.int/hiv/topics/en/HealthcareSeeking.pdf

[B3] Case A Menendez A Ardington C Health seeking behaviour in Northern KwaZulu-Natal CSSR Working Paper No. 116 2005 , Cape Town http://www.saldru.uct.ac.za/papers/cssrwps/wp116.pdf

[B4] Bradshaw D Groenewald P Laubscher R Nannan N Nojilana B Norman R Pieterse D Schneider M Bourne DE Timæus IM Dorrington R Johnson L Initial burden of disease estimates for South Africa, 2000 S Afr Med J 2003 93 682 688 14635557

[B5] Popkin BM An overview on the nutrition transition and its health implications: the Bellagio meeting Public Health Nutr 2002 5 1A 93 103 12027297 10.1079/phn2001280

[B6] Myer L Ehrlich RI Susser ES Social epidemiology in South Africa Epidemiol Rev 2004 26 112 123 10.1093/epirev/mxh004 15234952

[B7] Franzini L Caughy M Spears W Fernandez Esquer ME Neighborhood economic conditions, social processes and self-rated health in low-income neighbourhoods in Texas: A multilevel latent variables model Soc Sci Med 2005 61 6 1135 1150 10.1016/j.socscimed.2005.02.010 15970226

[B8] Statistics South Africa General household survey, 2010; 2010 http://www.statssa.gov.za/publications/P0318/P0318June2010.pdf

[B9] Cummings KM Becker MH Maile MC Bringing the models together: an empirical approach to combining variables used to explain health actions J Behav Med 1980 3 2 123 145 10.1007/BF00844986 7420418

[B10] Kroeger A Anthropological and socio-medical health care research in developing countries Soc Sci Med 1983 17 3 147 161 10.1016/0277-9536(83)90248-4 6836349

[B11] Rand health Medical Outcomes Study (MOS): 36-item short-form survey 2007 http://www.rand.org/health/surveys_tools/mos/mos_core_36item.html

[B12] Ware JE Sherbourne CD The MOS 36-item short-form health survey (SF-36): 1. Conceptual framework and item selection Med Care 1992 30 473 483 10.1097/00005650-199206000-00002 1593914

[B13] Rand health Scoring instructions for MOS 36-Item Short Form Survey Instrument (SF-36).:; 2007 http://www.rand.org/health/surveys_tools/mos/mos_core_36item_scoring.pdf

[B14] Norcross WA Ramirez C Palinkas LA The influence of women on the health care-seeking behavior of men J Fam Pract 1996 43 5 475 480 8917147

[B15] Banerjee A Galiani S Levinsohn J McLaren Z Woolard I Why Has Unemployment Risen in the New South Africa? In National Bureau of Economic Research (NBER) Working Paper No 2007 http://www.nber.org/papers/w13167

[B16] Delany A Ismail Z Graham L Ramkissoon Y Review of the Child Support Grant: uses, implementation and obstacles UNICEF 2008 http://www.info.gov.za/view/DownloadFileAction?id=90553

[B17] Kruger A Greeff M Watson MJ Fourie CMT Health care seeking behaviour of newly diagnosed HIV infected people from rural and urban communities in the North West Province of South Africa Afr J Nurs Midwifery 2009 11 2 30 47

[B18] Agyemang C Rural and urban differences in blood pressure and hypertension in Ghana, West Africa Public Health 2006 120 525 533 10.1016/j.puhe.2006.02.002 16684547

[B19] Hirschowitz R Taunyane L De Castro J Segel K Hirschowitz S A national household survey of health inequalities in South Africa Report by Community Agency for Social Enquiry (CASE) for the Henry J. Kaiser Family Foundation.:; 1995 http://www.healthlink.org.za/uploads/files/case.pdf

[B20] Pinkoane MG Greeff M Williams MJ The patient relationship and therapeutic techniques of the South Sotho traditional healer Curationis 2005 28 4 20 30 16450556 10.4102/curationis.v28i4.1005

[B21] Khun S Manderson L Health seeking and access to care for children with suspected dengue in Cambodia: an ethnographic study BMC Publ Health 2007 7 262 271 10.1186/1471-2458-7-262 PMC2164964 17892564

[B22] Ahmed SM Tomson G Petzold M Kabir ZN Socio-economic status overrides age and gender in determining health-seeking behaviour in rural Bangladesh Bull World Health Organ 2005 83 2 109 117 15744403 PMC2623805

[B23] Tanser F Gijsbertsen B Herbst K Modelling and understanding primary health care accessibility and utilization in rural South Africa: an exploration using a geographical information system Soc Sci Med 2006 63 691 705 10.1016/j.socscimed.2006.01.015 16574290

[B24] Belli P Gotsadze G Shahriari H Out-of-pocket and informal payments in health sector: evidence from Georgia Health Policy 2004 70 109 123 10.1016/j.healthpol.2004.03.007 15312713

[B25] Gotsadze G Bennett S Ranson K Gzirishivili D Health care-seeking behaviour and out-of-pocket payment in Tbilisi, Georgia Health Policy Plan 2005 20 4 232 242 10.1093/heapol/czi029 15965035

[B26] Collinson MA Tollman SM Kahn K Migration, settlement change and health in post-apartheid South Africa: triangulating health and demographic surveillance with national census data Scand J Public Health 2007 35 Suppl 69 77 84 10.1080/14034950701356401 PMC2830108 17676507

